# Tweeting for Success: The Role of Twitter in Enhancing SCImago Journal Rank for Specialty Surgical Journals

**DOI:** 10.7759/cureus.40867

**Published:** 2023-06-23

**Authors:** Heli Patel, Justin M Camacho, Saket Pandit, Gabriella Morey, Davek Sharma, Michael Karon, Amir Behnam

**Affiliations:** 1 Department of Medical Education, Nova Southeastern University Dr. Kiran C. Patel College of Allopathic Medicine, Davie, USA; 2 Department of Medicine, Drexel University College of Medicine, Philadelphia, USA; 3 Plastic and Reconstructive Surgery, Tower Health Medical Group, Reading, USA; 4 Plastic Surgery, Tower Health Medical Group, Reading, USA

**Keywords:** sjr, twitter, social media, impact factor, surgical journal

## Abstract

Background

The cornerstone of surgical education and practice is the surgical journal, but the ability to disperse the vital information within their pages had previously been limited. The use of Twitter by surgical journals has increased in recent years and has allowed these journals to reach a wider audience than they previously could. In this article, we discuss the role Twitter engagement has on a journal’s impact factor, visibility, and prestige.

Methods

The authors compiled a list of journals using the SciMago Journal and Country rank platform. Included journals’ Twitter profiles were then assessed using Twitonomy, an online platform that collects and processes data regarding individual Twitter profiles. Statistical analysis was conducted to determine the relationship between Twitter use and SCImago Journal Rank (SJR).

Results

Simple linear regression and multiple linear regression models determined that the only variables that had a statistically significant impact on all journals were the age of the Twitter account (p=0.003) and the percentage of retweets (p=0.001). When it comes to specialty-specific journals, further analysis showed that the only significant factor regarding its impact on SJR was the percentage of retweets (p=0.007).

Conclusions

Surgical journals' regular use of Twitter is important in the dissemination of important information to a wide audience. This article shows that the most important variable to determine the impact and visibility of a surgical journal is the percentage of retweets. Further research should be performed to better understand how to use Twitter and other social media platforms to reach a larger audience.

## Introduction

Since their advent, surgical journals have been a powerhouse of medical information. However, the manner in which surgical journals are advertised and obtained by the user has inevitably changed alongside the evolution of technology. From a historical perspective, awareness of the journals and their literature content depended on two things: (1) journal access and (2) the user’s knowledge regarding the journal’s platform to extract required information [1].

Traditionally, the merit and impact of a journal or article are determined based on bibliometrics [2]. Bibliometrics is a quantitative method of citation and content analysis of scholarly journals, books, and researchers [3]. In recent years, social media has changed the way in which medical knowledge is disseminated. With a tenfold growth over the past decade, social media has the ability to reach a larger and broader audience through an active form of marketing [4]. As a result of the influential and powerful nature of social media, a journal’s presence on social media will undoubtedly impact its ability to capture its targeted audience and scholarly ranking.

To date, Twitter remains unparalleled as a leader in the transmission of evidence-based information to members of the medical community [1]. Surgical journals have utilized the Twitter platform to generate greater engagement between the medical community and individual Twitter users by taking advantage of the various tools used in a simple tweet, i.e., hyperlinks, infographics, visual abstracts, and text [5]. The development and maintenance of long-term connections with users allow the medical community to stay actively engaged in their respective fields while delving into the latest trends and scientific developments. Through this platform, users are not only exposed to the scientific marvels of their field but are inspired to take part in the transformation of their respective specialties.

Despite the popularity and ubiquitous nature of Twitter, a limited number of studies examine how Twitter usage may affect the impact of the journals' scientific influence as measured by the SCImago Journal Rank (SJR) [6]. The SJR is a publicly available portal that serves to measure a journal’s impact, influence, or prestige as it expresses the average number of weighted citations received in a given year by the documents published in a selected journal in the previous three years [7-13]. To address the gap in the current literature, our study evaluates how a journal's SJR index was affected by variables indicative of a profile's Twitter engagement.

## Materials and methods

Utilizing the SCImago Journal and Country Rank platform, a search was conducted on May 15, 2022, to identify relevant surgical journals in the following specialties: plastic surgery, cardiac surgery, orthopedic surgery, and neurosurgery. To ensure all relevant journals were accounted for, we utilized the following MeSH terms: "plastic surgery," "plastic and reconstructive surgery" and "aesthetic surgery," "thoracic" and "cardiac surgery" and "cardiovascular surgery," "orthopaedic" and "orthopedic," and "neurosurgery." The aforementioned MeSH terms were utilized in conjunction with the journal category filter tool provided by the SCImago platform to further validate our screening process. Journals were excluded on the basis of either non-English language or lack of presence on Twitter. Furthermore, journals with Twitter accounts associated with multiple specialties were excluded as well. Of the 86 journals found, 54 were included based on our inclusion criteria. For journals with dedicated Twitter profiles, the change in SJR between the year the Twitter account was established and 2021 was recorded. Whether or not journals experienced an increase in SJR since joining Twitter was investigated.

Assessment of the journal's Twitter involvement was assessed utilizing the Twitonomy software, an online platform that allows users to collate and process data related to individual user profiles. Utilization of this software was found in a study that performed a similar analysis solely in the field of orthopedics [6]. Through the Twitonomy platform, we collected the relevant account information for each journal, from the time of its Twitter creation up until December 2021. Journals that joined Twitter in 2020 were excluded due to a lack of measurable change in the SJR. Quantification of a surgical journal's Twitter presence was standardized utilizing the following variables since creation: the number of followers, the total number of tweets, the percentage of retweets, and the age of the account in years. Additionally, this study also investigated how these variables differ by the medical specialty that a journal falls under (Journal Specialty). All information used in this analysis is accurate as of May 15, 2022.

Statistical analysis was conducted using Student’s t-test (one-sample), analysis of variance (ANOVA), and Pearson correlations. All statistical analysis was performed using R Software version 4.1.3 (2022-03-10). Prior to any analysis, all values were normalized so that they could more accurately be compared despite spanning over different ranges. This analysis was performed at two levels: first, by looking at associations between Twitter presence and SJR ranking across specialties and then by looking at these associations on a specialty-by-specialty basis.

## Results

To explore and understand the relationship between Twitter presence and SJR ranking, several statistical methods were employed to assess the statistical importance of our variables of interest. These included simple linear regression models, multiple linear regression models, and the ANOVA procedure.

This analysis was performed at two levels: first, by looking at associations between Twitter presence and SJR ranking across specialties; second, by looking at these associations on a specialty-by-specialty basis. The analysis that includes all specialties is outlined in the Global Analysis section, and the specialty-by-specialty analysis is outlined in the Analysis by Specialty section.

Global analysis

Single Variable Linear Regressions

**Figure 1:**
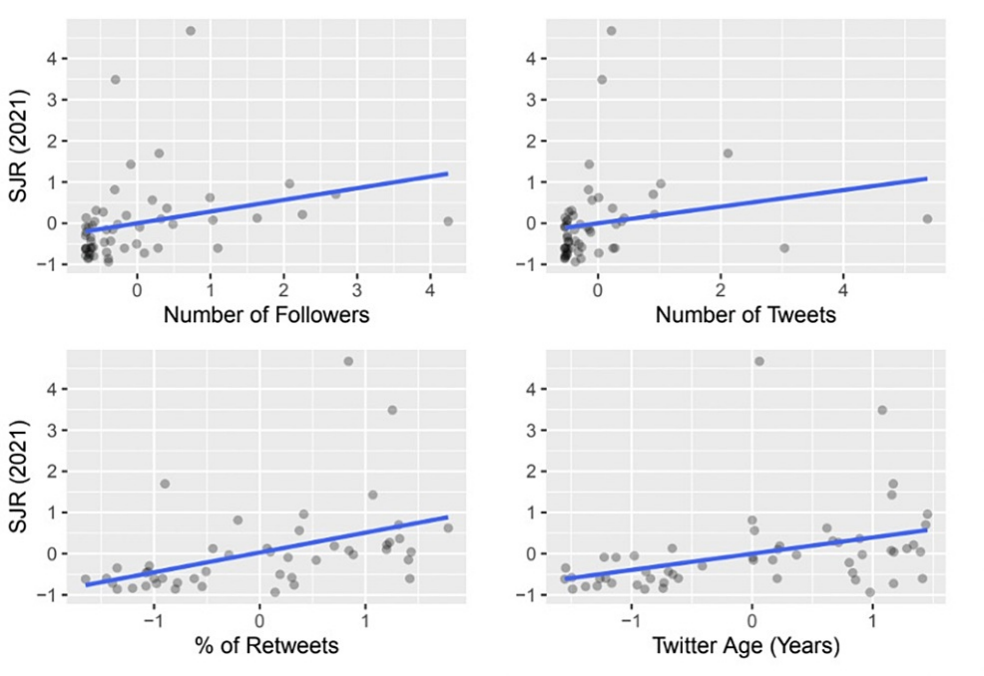
Analysis of individual Twitter factors in relation to SJR The graphical representation shows the positive relationship each factor has on journals’ SJR, but only % of retweets and Twitter age showed a statistically significant response.

In this analysis, each of the variables mentioned above was used singly as a predictor to model the SJR score via a simple linear regression. The global analysis results are visualized in Figure 1 and summarized in Table 1.

**Table 1:** Twitter variables analyzed to assess the impact on SJR All four Twitter variables exhibit a positive relationship on journals’ SJR, but only % of retweets and Twitter age showed a statistically significant response.

	Estimate	R-squared	p-value
Number of Followers	0.284	0.08	0.038
Number of Tweets	0.201	0.041	0.144
Percentage of Retweets	0.481	0.204	0.001
Twitter Age (Years)	0.394	0.155	0.003

Table 1 shows each single-variable regression’s parameter estimate, coefficient of determination (R-squared), and p-value (P) across all surgical specialties in consideration. In order to account for multiple hypothesis tests, the p-values generated by these regressions were evaluated for significance using the Bonferroni correction. This correction decreases the threshold for significance by taking into account the number of hypothesis tests being performed. Therefore, a confidence level of α = 0.05/4 = 0.0125 was used.

As shown in Table 1, the hypothesis test for each regression coefficient was that including the variable in the model is better than the absence of the variable. Utilizing the Bonferroni corrected level of confidence of 0.0125, the only variables that are significantly associated with the SJR score of a journal are the percentage of retweets and Twitter age (years). Additionally, the positive parameter estimate of these variables suggests a positive correlation between the aforementioned variables and the SJR score. This means that, when considered by itself, an increase in the percentage of a journal account’s tweets that are retweeted is associated with an increase in the journal’s SJR score. A synonymous phenomenon was found when we analyzed the relationship between the journal's account age and the SJR score.

Multiple Linear Regression

Based on the results from the global analysis in Table 1, the percentage of retweets and Twitter age (years) were regressed against the SJR for a journal via multiple linear regression. The results of the regression are reported in Table 2. For this regression, R2-adjusted = 0.200, and P = 0.003.

**Table 2:** Subsequent multivariate analysis for significant covariates Based on the results in Table 1, the percentage of retweets and Twitter age (years) were regressed against the SJR for a journal via multiple linear regression. Neither of these variables was significantly correlated with the SJR score.

	Estimate	p-value
Percentage of Retweets	0.334	0.064
Twitter Age (Years)	0.239	0.182

As shown in the regression model in Table 2, the Bonferroni correction yields alpha = 0.05/2 = 0.025. Utilizing the corrected Bonferroni confidence level, the p-values of the parameter estimates suggest that neither of these variables are significantly correlated with the SJR score. This is an interesting result, as both percentages of retweets and Twitter age (years) were highly significant when regressed individually. Furthermore, it appears that the effect of each variable’s association with the SJR is diminished when both variables are present in the model, as indicated by the decrease in the parameter estimates when compared to the single-variable analysis shown in Table 1. This may mean that there is some confounding present between these variables; however, further investigation yielded an insignificant interaction term (Supplemental Figure x). Instead, these results suggest that each variable accounts for the same variance in the SJR score. Therefore, either percentage of retweets or Twitter age (years) can be used to explain the variation between the journal’s SJR scores.

Analysis by specialty

Differences in Means Between Specialties

In order to determine if there was a significant difference in SJR scores when taking the journal specialty into consideration, an ANOVA test was performed. Implementation of the ANOVA test allowed us to determine whether there is a significant difference in the mean of the SJR when the independent variables are taken into account, individually and collectively. This type of analysis is beneficial when the effects of categorical variables are being investigated, as is the case with the journal specialty variable. The results of the ANOVA test are shown in Table 3.

**Table 3:** ANOVA analysis (specialty specific) Only the percentage of retweets emerges as significant for SJR changes when the independent variable is analyzed while controlling all other variables.

	Df	Sum Sq	Mean Sq	f-value	Pr (>F)
Number of Followers	1	3.905	3.905	7.882	0.009
Number of Tweets	1	0.405	0.405	0.818	0.374
Percentage of Retweets	1	6.839	6.839	13.805	0.001
Twitter Age (Years)	1	1.082	1.082	2.184	0.151
Journal Specialty	3	5.883	1.961	3.958	0.018
Number of Followers: Journal Specialty	3	16.125	5.375	10.85	0.000
Number of Tweets: Journal Specialty	3	1.937	0.646	1.304	0.294
Percentage of Re-tweets: Journal Specialty	3	1.193	0.398	0.803	0.503
Twitter Age (Years): Journal Specialty	3	1.284	0.428	0.864	0.472
Residuals	27	13.376	0.495	NA	NA

Using the Bonferroni correction for multiple tests of significance, we evaluated the effect of these variables at the α = 0.05/9 = 0.006 significance level. A significant p-value for this analysis indicates that the SJR changes with the independent variable under consideration when all other variables are controlled for. In this analysis, only the percentage of retweets emerges as significant. This is consistent with the results of the former analysis shown in Table 2, in which the percentage of retweets was significantly correlated with SJR scores.

Intriguingly, it also appears that the number of followers and journal specialty may also be significant. This is indicated by their relatively low p-value when considered individually and significant interaction terms. The effects of these variables in relation to the SJR of a journal are the subject of the subsequent analysis.

The results of the previous ANOVA analysis (Table 3) underscore which variables to include in the multiple linear regression. Consequently, we regressed the SJR score against the number of followers, percentage of retweets, and journal specialty. The results of this regression are summarized in Table 4. For this regression, R2-adjusted = 0.225, and P = 0.008.

**Table 4:** Multivariate regression analysis based on the specialty of journals When the specialty of the journal is taken into account, only the percentage of a journal’s tweets that get retweeted shows a positive correlation with the SJR of a journal.

	Estimate	p-value
Number of Followers	0.152	0.327
Percentage of Retweets	0.441	0.007
Journal Specialty - Cardiothoracic Surgery	0.43	0.11
Journal Specialty - Neurosurgery	0.173	0.576
Journal Specialty - Orthopedic Surgery	-0.173	0.482
Journal Specialty - Plastic Surgery	-0.485	0.151

## Discussion

The results of this study suggest that there is a relationship between a journal’s Twitter activity and its SJR score. This is consistent with previous studies that have also found an association between Twitter activity and SJR, specifically with the number of followers [10,11,14-16]. However, these studies only looked within one specialty, not across multiple specialties as in this analysis.

Specific to our analysis, the percentage of retweets emerged as having the most significant relationship with SJR out of all the variables assessed. The percentage of retweets was consistently associated with a statistically significant p-value, either through single linear regressions or multiple linear regression. Intriguingly, the percentage of retweets remained significant even when the journal specialty was controlled for, which could mean that this relationship remains constant across different medical specialties. Future studies into the relationship between Twitter activity and journal impact should look more into the effect that the percentage of retweets has on predicting a journal’s impact.

Previous studies have investigated the impact that other social media, such as Facebook or Instagram, have on a journal’s impact [17-21]. All of these studies have shown that engagement on social media is predictive of journals having a greater impact. This effect is constant regardless of the type of social media under investigation. The novel contribution of this investigation is that we look into the specific factors that are most predictive of higher impact. Future studies can look into whether these trends hold across other social media platforms, such as TikTok or Instagram.

Some limitations of this study include the sample size of journals under consideration. This study only analyzed 58 journals, of which only 54 had Twitter metrics available. Furthermore, our study only looked at journals from four different medical specialties. This limits the statistical power of the effects noted in our investigation. Future studies can benefit from investigating whether this effect remains consistent when considered over a larger number of journals and across multiple different specialties. Lastly, the granularity of the data was also a limiting factor. Our data only included Twitter data as of May 15, 2022. Future studies can look into dynamic relationships between Twitter activity and SJR scores and how the relationship between these variables changes over time.

## Conclusions

In recent years, surgical specialties have increased utilization of Twitter as it relates to their impact on the medical community. Overall, in our study, a significant relationship was observed between the journal’s SJR and Twitter activity along with the respective surgical specialty. At every stage of analysis, the percentage of retweets was found to have the greatest significance of all variables analyzed in this study. Given the exponential use of social media and its growing presence in the field of medicine, it is imperative that further research is conducted to understand the interconnectedness of social media and journal impact factors. Furthermore, understanding the link between social media presence and its effects on capturing the intended audience will allow new scientific developments to reach greater distances that will inevitably impact the care delivered to patients around the globe.
